# Behaviors of AlGaN Strain Relaxation on a GaN Porous Structure Studied with d-Spacing Crystal Lattice Analysis

**DOI:** 10.3390/nano13101617

**Published:** 2023-05-11

**Authors:** Hao-Yu Hsieh, Ping-Wei Liou, Shaobo Yang, Wei-Cheng Chen, Li-Ping Liang, Yueh-Chi Lee, Chih-Chung (C. C.) Yang

**Affiliations:** Institute of Photonics and Optoelectronics, and Department of Electrical Engineering, National Taiwan University, No. 1, Section 4, Roosevelt Road, Taipei 10617, Taiwan; r08941076@ntu.edu.tw (H.-Y.H.); r08941086@ntu.edu.tw (P.-W.L.); f08941106@ntu.edu.tw (S.Y.); r09941091@ntu.edu.tw (W.-C.C.); r09941092@ntu.edu.tw (L.-P.L.); r09941110@ntu.edu.tw (Y.-C.L.)

**Keywords:** GaN porous structure, strain relaxation, d-spacing crystal lattice analysis, strain-damping, critical thickness

## Abstract

The high porosity of a GaN porous structure (PS) makes it mechanically semi-flexible and can shield against the stress from the thick growth template on an overgrown layer to control the lattice structure or composition within the overgrown layer. To understand this stress shield effect, we investigated the lattice constant variations among different growth layers in various samples of overgrown Al_0.3_Ga_0.7_N on GaN templates under different strain-relaxation conditions based on d-spacing crystal lattice analysis. The fabrication of a strain-damping PS in a GaN template shields against the stress from the thick GaN template on the GaN interlayer, which lies between the PS and the overgrown AlGaN layer, such that the stress counteraction of the AlGaN layer against the GaN interlayer can reduce the tensile strain in AlGaN and increase its critical thickness. If the GaN interlayer is thin, such that a strong AlGaN counteraction occurs, the increased critical thickness can become larger than the overgrown AlGaN thickness. In this situation, crack-free, thick AlGaN overgrowth is feasible.

## 1. Introduction

The strain built in a GaN template controls the strain condition and hence the crystal quality of an overgrown nitride layer, which is critical for device fabrication. This issue is particularly important when a quantum-well (QW) structure is overgrown for light-emission application. For instance, the indium incorporation efficiency of an overgrown InGaN/GaN QW and hence its emission wavelength and efficiency are greatly influenced by the compressive strain caused by the thick GaN template used for QW overgrowth [1]. Moreover, the crystal quality of an overgrown AlGaN layer is strongly affected by the tensile strain resulting from its thick GaN template. A strong tensile strain in the AlGaN layer leads to a small critical thickness such that this layer can be easily cracked [2]. Hence, if the strain in the template can be significantly released, we can more easily control the strain condition in the overgrown layer to improve its crystal quality. For this purpose, the fabrication of a porous structure (PS) to shield against the strain from the thick template is an attractive technique.

A layer of heavily Si-doped GaN, which is usually referred to as an n^+^-GaN layer, can be easily etched with acids, such as HNO_3_, or alkalis, such as KOH, as electrolytes in an electrochemical etching (ECE) process to form a PS [3,4,5,6,7,8]. In this process, the etching direction essentially follows the flow of electric current for forming subsurface lateral pores in GaN. Many applications based on GaN or AlGaN PSs have been explored, including the improvements of LED crystal quality and light extraction [9,10,11,12,13], the liftoff of a GaN layer or an LED structure from its substrate [14,15,16], the enhancement of water-splitting efficiency [17,18], the formation of a distributed Bragg reflector [19,20], the increase in sensor sensitivity [21], the relaxation of strain [22], and the fabrications of lateral and vertical laser diodes [23,24,25]. More applications are expected to be developed based on such a subsurface PS.

Recently, the overgrowth of AlGaN or InGaN on a GaN PS has attracted much research attention [12,13,26,27]. Such an overgrowth has the advantage that the GaN PS layer is mechanically semi-flexible such that it can damp or shield against the stress from the GaN template on the overgrown layer. Therefore, the overgrown layer can become almost strain-free if its thickness is significantly larger than that of the capping GaN layer, i.e., the GaN layer above the PS. Taking AlGaN overgrowth on GaN as an example, when the AlGaN layer is thicker than its critical thickness, the tensile strain in AlGaN relaxes and this layer may crack [2]. Typically, a stronger tensile strain results in a more dramatic strain relaxation or a smaller critical thickness and hence a stronger cracking behavior. When a strain buffer layer, such as a GaN PS, exists in the GaN template, the strain effect from the GaN template on the overgrown AlGaN becomes weaker. In this situation, the tensile strain in AlGaN becomes weaker, leading to a larger critical thickness or a weaker cracking behavior. It has been demonstrated that crack-free AlGaN of 15% in Al content and 1.3 μm in thickness could be overgrown with metalorganic chemical vapor deposition (MOCVD) on a GaN template with a PS [26]. In this effort, to further enhance strain relaxation, a mesa array was fabricated. The same concept has also been applied to the overgrowth of InGaN on a GaN PS template. Based on molecular beam epitaxy (MBE), InGaN of 20% in In content has been overgrown on such a pseudo-substrate. The compressive strain relaxation in the InGaN layer can be as large as 60% [12]. It was claimed that the surface of this InGaN layer was free of V-shaped defects, and the threading dislocation density was comparable to that in the GaN template. By overgrowing an InGaN layer on a GaN PS template with a 10 μm × 10 μm mesa array, the InGaN emission wavelength can be red-shifted from 506 to 547 nm [27]. Based on this technique, an InGaN micro-LED of <10 μm in mesa size and 632 nm in emission wavelength has been implemented to show >0.2% on-wafer external quantum efficiency [13].

Although the use of a GaN PS as a strain damper for overgrowing an AlGaN layer of a weaker tensile strain has been proposed and demonstrated [26], the strain variation along crystal growth in such a sample structure has not been well studied. The understanding of such strain variation behaviors is important for properly designing the n^+^-GaN structure and the GaN capping layer such that the strain condition in the overgrown AlGaN layer can be well controlled. To investigate the layer-by-layer strain variation in such a sample, a microscopic method is useful. In this paper, we report the results of such an investigation based on d-spacing crystal lattice analysis [28]. From the analyses of atomic-scale transmission electron microscopy (TEM) images, we can obtain the lattice constants in different GaN and AlGaN layers and observe their variations along crystal growth. In Section 2 of this paper, the sample structures, their fabrication conditions, and their characterization methods are described. Then, in Section 3, by using one of the samples as an example, we demonstrate the detailed characterization procedures and results. Next, the lattice constant data in all samples under study are summarized and discussed in Section 4. Further discussions about the observed results are made in Section 5. Finally, conclusions are drawn in Section 6.

## 2. Sample Structures, Fabrication Conditions, and Characterization Methods

**Figure 1:**
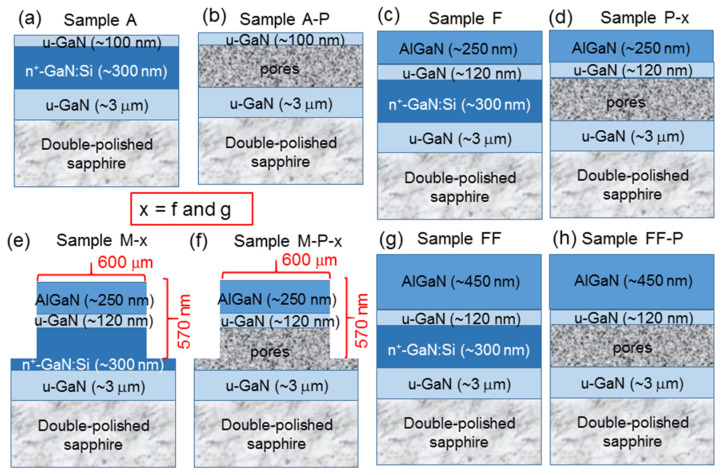
(**a**–**h**): Schematic illustrations of the structures in various samples.

For this study, we prepared nine AlGaN-on-GaN samples to compare their lattice constants or strain relaxation conditions in different layers. Each of those samples was obtained by overgrowing an AlGaN layer of ~30% in Al content and ~250 or ~450 nm in thickness with MBE on a GaN template, which contains an n^+^-GaN layer of ~300 nm in thickness for fabricating a PS through an ECE process. As schematically illustrated in Figure 1a for the structure of the GaN template, which is designated as sample A, a ~3 μm undoped GaN (u-GaN) with the full-width at half-maximum of its rocking curve at 228 arcsec is first grown on a double-polished c-plane sapphire substrate based on MOCVD. Then, the 300 nm n^+^-GaN layer of 2 × 10^19^ cm^−3^ in Si doping concentration is deposited, followed by a u-GaN capping layer of ~100 nm in thickness. Either an n^+^-GaN or a u-GaN layer is grown at a temperature of 1040 °C in the MOCVD chamber. To understand the strain-relaxation behavior in sample A when a PS is fabricated in the n^+^-GaN layer, we prepared sample A-P, as schematically illustrated in Figure 1b. Before the overgrowth of AlGaN with MBE, a ~20-nm GaN is deposited onto the GaN template, i.e., sample A, in the MBE chamber. Therefore, as illustrated in Figure 1c for sample F, between the n^+^-GaN and AlGaN layers, the total thickness of the u-GaN interlayer is ~120 nm. The MBE growth conditions for the GaN and AlGaN layers include substrate temperature at 758 °C, Ga effusion cell temperature at 1028 (993) °C for GaN (AlGaN) growth, Al effusion cell temperature at 1035 °C, nitrogen flow rate at 0.6 sccm, RF plasma power at 200 W, and a growth duration of 20 min for the GaN layer. The growth duration for the AlGaN layer of ~250 (~450) nm in thickness is 300 (540) min.

To observe the strain-relaxation behaviors of AlGaN, we prepared samples with either a PS, a mesa structure, or both through two different procedures. As illustrated in Figure 1d for sample P-x with x = f (g), we fabricated the PS (overgrow the AlGaN layer) first and then overgrew the AlGaN layer (fabricated the PS) to yield sample P-f (P-g). Similarly, as illustrated in Figure 1e for sample M-x with x = f (g), we fabricated the mesa structure (overgrew the AlGaN layer) first and then overgrew the AlGaN layer (fabricated the mesa structure) to produce sample M-f (M-g). The PS was fabricated under ECE conditions of 12 V in applied voltage, 5 wt% in HNO_3_ concentration (the electrolyte), and 15 min in etching duration. To prepare sample M-g, after AlGaN overgrowth, we used the techniques of photolithography and inductively coupled plasma reactive ion etching (ICPRIE) to fabricate a square-mesa array of 600 μm in mesa dimension, 300 μm in edge-to-edge mesa spacing, and ~570 nm in mesa height, as illustrated in Figure 1e. To prepare sample M-f, a mesa pattern was fabricated first on the GaN template (sample A) with an etching depth at ~300 nm. After the ~20 nm GaN and ~250 nm AlGaN layers were overgrown, the second process of lithography/ICPRIE was undertaken with the etching depth at ~270 nm to remove the overgrown portion outside the mesas, leading to the final mesa height still around 570 nm. By fabricating a PS under the same ECE conditions in sample M-g, we obtained sample M-P-g, with the structure schematically illustrated in Figure 1f. To prepare sample M-P-f, AlGaN overgrowth and then the second lithography/ECE process were undertaken after the mesa pattern and PS were fabricated. The strain-relaxation mechanisms in sample M-P-f or M-P-g include both PS and mesa structure. As illustrated in Figure 1g, the structure of sample FF is the same as that of sample F except that the overgrown AlGaN thickness is ~450 nm in sample FF. By fabricating a PS in sample FF based on the same ECE conditions, we obtained sample FF-P, as illustrated in Figure 1h.

**Figure 2:**
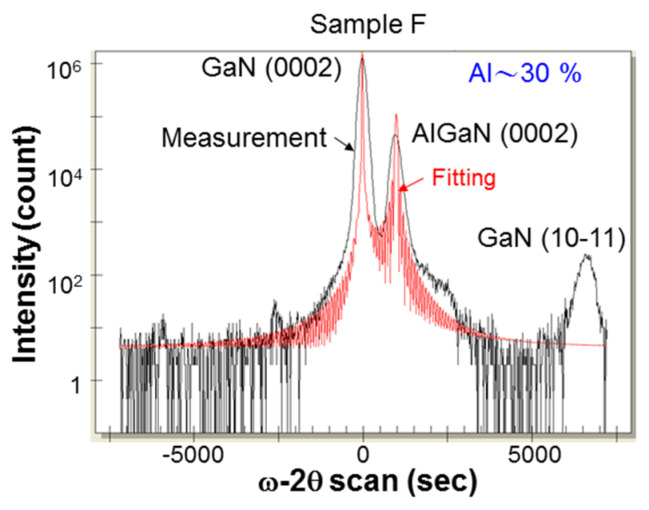
Results of (0002)-plane ω-2θ scan in XRD measurement for sample F. From fitting, we estimated the Al content in the AlGaN layer to be ~30%.

To estimate the Al composition in an AlGaN layer overgrown with the designated MBE conditions, a fundamental X-ray diffraction (XRD) measurement was undertaken (using the facility of model D1 manufactured by Bede). Figure 2 shows the (0002)-plane ω-2θ scan result of the XRD measurement for sample F, which contained an overgrown AlGaN layer of ~250 nm in thickness on a GaN template without PS. By fitting the ω-2θ scan result based on a built-in software in the XRD facility (red curve in Figure 2), we estimated the Al content in this sample to be ~30%. Since the MBE growth conditions for AlGaN were the same in all AlGaN samples under study, their Al contents were expected to be about the same. Certain small variations may have existed because of the different strain conditions in the growth templates. However, we will use an Al content of 30% for the following discussions about the lattice constant variations among different samples.

**Figure 3:**
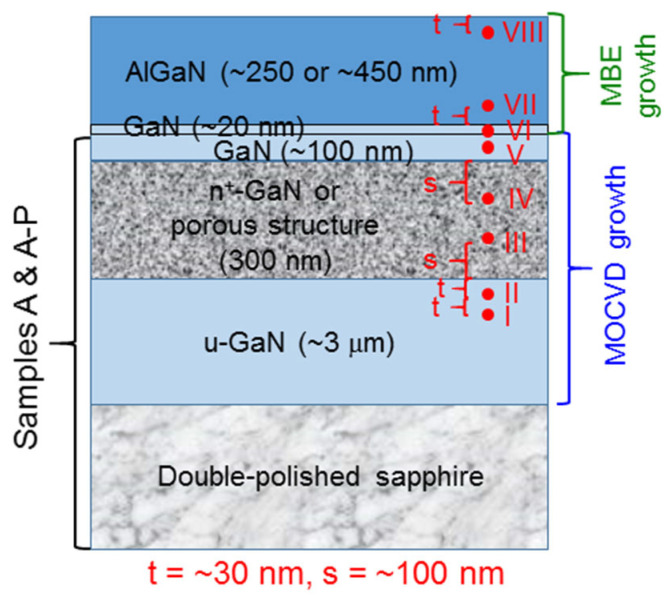
Schematic illustration of the five or eight locations for d-spacing crystal lattice analysis.

In this research, we used d-spacing crystal lattice analysis to analyze the atomic-scale TEM images (using model JEM-ARM200FTH manufactured by JEOL) in order to understand the lattice size variations in different layers of the samples under study. The details of this method will be demonstrated in Section 3 by using sample M-P-f as an example. To compare the lattice constants among different samples, the cross-sectional locations for d-spacing analysis were chosen to be the same in different samples. As schematically illustrated in Figure 3, eight locations (locations I–VIII) were designated in different layers for a sample with AlGaN overgrowth. For either sample A or A-P, only five locations (locations I–V) were designated for d-spacing analysis. As illustrated in Figure 3, locations I and II are situated in the ~3 μm u-GaN layer at the vertical distances of 2t and t (t = ~30 nm), respectively, from the lower boundary of the n^+^-GaN layer. Locations III and IV are situated in the n^+^-GaN or PS layer with the vertical distances from the lower and upper boundaries, respectively, of this layer at s = ~100 nm. Location V is situated in the middle of the ~100 nm capping u-GaN layer grown with MOCVD. Location VI is situated in the middle of the ~20 nm u-GaN layer overgrown with MBE. Finally, locations VII and VIII are situated in the overgrown AlGaN layer with the vertical distances from the lower and upper boundaries, respectively, of this layer at t = ~30 nm. The square dimension of an atomic-scale TEM image for a d-spacing analysis location is ~35 nm.

## 3. Demonstrations of Characterization Procedures and Results Using Sample M-P-f

**Figure 4:**
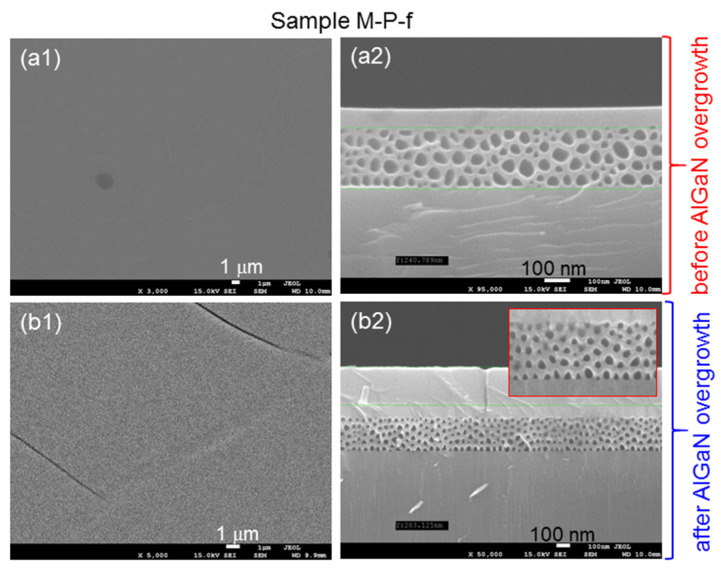
(**a1**,**a2**): Plane-view and cross-sectional SEM images, respectively, before AlGaN overgrowth in sample M-P-f. (**b1**,**b2**): Plane-view and cross-sectional SEM images, respectively, after AlGaN overgrowth and removal of the AlGaN layer outside the mesa regions in sample M-P-f. The inset in (**b2**) shows a magnified portion of the PS after AlGaN overgrowth.

**Figure 5:**
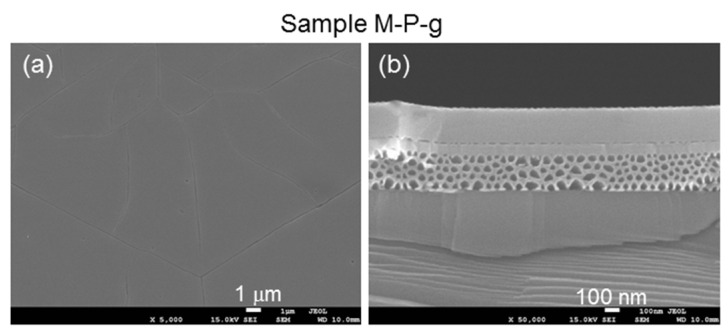
(**a**,**b**): Plane-view and cross-sectional SEM images, respectively, after AlGaN overgrowth and the fabrications of the PS and the mesa structure in sample M-P-g.

In this section, we use sample M-P-f as an example to demonstrate the measurement and analysis procedures. Figure 4(a1,a2) show the plane-view and cross-sectional scanning electron microscopy (SEM) images, respectively, of sample M-P-f after mesa and PS fabrications, but before AlGaN overgrowth (using model JSM-7001F manufactured by JEOL). We can see that the sample surface is quite smooth. Also, the whole n^+^-GaN layer has been etched to form a PS layer of ~300 nm thickness. The cross-sectional sizes of the pores range from 30 to 100 nm in Figure 4(a2). Figure 4(b1,b2) show the plane-view and cross-sectional SEM images, respectively, of sample M-P-f after AlGaN overgrowth. In Figure 4(b1), we can see cracking lines on the surface of the overgrown AlGaN layer, indicating the strain-relaxation result. The critical thickness of Al_0.3_Ga_0.7_N grown on a solid sapphire-based GaN template was estimated to be only a few tens of nm [2]. The overgrown AlGaN thicknesses in the samples in the current study are significantly larger than this critical thickness. In Figure 4(b2), one can see that after AlGaN/GaN overgrowth with the substrate temperature at 758 °C for 320 min, the pores in the PS shrink. Here, the cross-sectional sizes of the pores range from 20 to 50 nm. By comparing a selected portion of the after-overgrowth SEM image shown in the inset of Figure 4(b2), which has the same magnification as that in Figure 4(a2), with the PS image before AlGaN/GaN overgrowth, we can indeed observe the shrunken nano-pores through the heating effect during overgrowth. For comparison, in Figure 5a,b, we show the plane-view and cross-sectional SEM images of sample M-P-g, for which the AlGaN layer is grown before the fabrications of the PS and mesa structure. Here, cracking lines can also be observed on the sample surface. The pore size in this PS (30–100 nm) is about the same as that in sample M-P-f before AlGaN overgrowth [see Figure 4(a2)].

**Figure 6:**
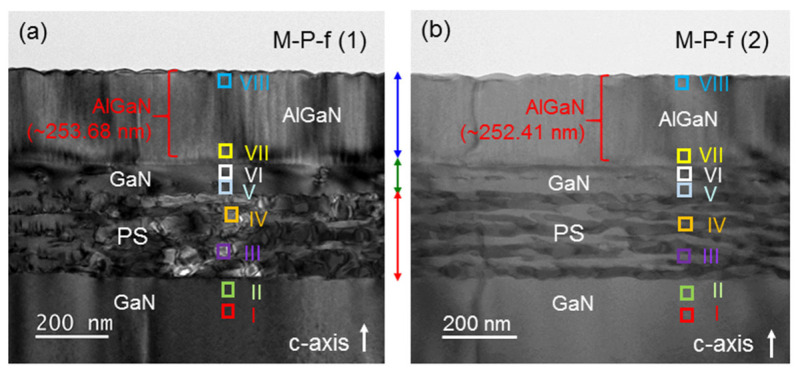
(**a**,**b**): TEM images in regions (1) and (2), respectively, of d-spacing crystal lattice analysis in sample M-P-f. In each image, the eight locations for d-spacing analysis are indicated by the squares.

**Figure 7:**
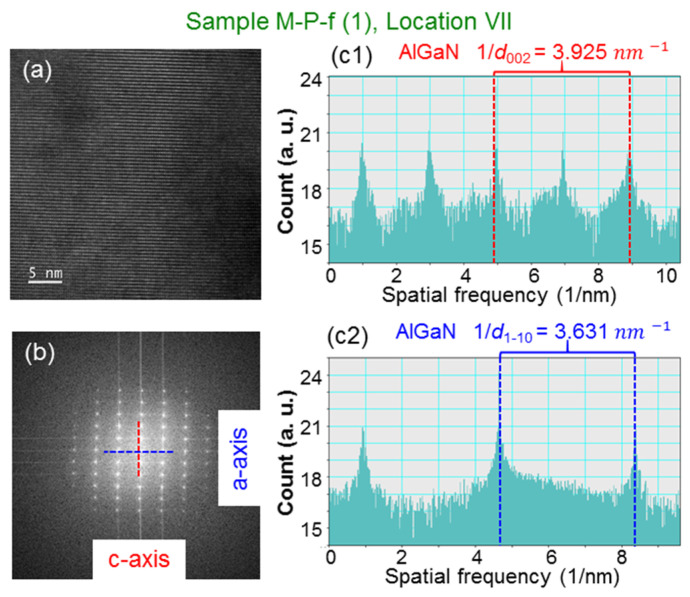
(**a**) Atomic-scale TEM image at location VII in region (1) of sample M-P-f for d-spacing analysis. (**b**) Lattice diffraction pattern after two-dimensional Fourier transform of the TEM image in (**a**). (**c1**,**c2**) Line-scan pattern along the c- [a-] axis of the lattice diffraction pattern in (**b**), from which we can evaluate 1/d_002_ [1/d_1–10_] to give 1/d_002_ = 2/*c* = 3.925 nm^−1^ [1/d_1–10_ = $2/\sqrt{3}a$ = 3.631 nm^−1^].

Figure 6a,b show the cross-sectional TEM images at two positions in sample M-P-f. The magnifications of the two images are adjusted to be about the same. The eight locations for d-spacing analysis are marked in both TEM images. By selecting location VII in the TEM image shown in Figure 6a, i.e., M-P-f (1), as an example, in Figure 7, we demonstrate the procedure of d-spacing analysis. In Figure 7a, we show the high-resolution TEM image at this location. After two-dimensional Fourier transform of this atomic-scale image, we can obtain the diffraction pattern in Figure 7b. Then, Figure 7(c1,c2) shows the line-scan intensity profile along the red- [blue-] dashed line in Figure 7b, which is oriented along the c- [a-] crystal axis. In Figure 7(c1,c2), we mark the central major peak and the second [first] minor peak on the right with vertical dashed lines for evaluating their spatial frequency separation to give 1/d_002_ = 3.925 nm^−1^ [1/d_1–10_ = 3.631 nm^−1^], which is equal to 2/*c* $[2/\sqrt{3}a$]. Here, *c* (*a*) is the lattice constant along the c- [a-] axis. From the d-spacing analysis, we can obtain *c* = 0.5096 nm and *a* = 0.3180 nm for AlGaN at location VII in sample M-P-f. Such an analysis procedure was applied to all designated analysis locations of the two sample positions in all samples under study. Table 1 shows the analysis results of lattice constants *a* and *c* at the eight locations in the two TEM images [(1) and (2)] of sample M-P-f. Here, one can see that for either lattice constant *a* or *c*, the two sets of data obtained from two different sample positions are highly consistent. In particular, the two sets of data for lattice constant *a* are almost identical. For lattice constant *c*, although the differences between the two sets of data at the deep locations are relatively larger, they are quite small at the shallow locations. The consistencies between the two sets of data indicate that the use of four digits after the decimal points is meaningful. In Table 1, we also show the average values and error ranges (the numbers after the ± sign) based on the two sets of data at each d-spacing analysis location. The results in Table 1 and those from other samples will be used for further discussions below.

## 4. Results of Strain Relaxation

**Figure 8:**
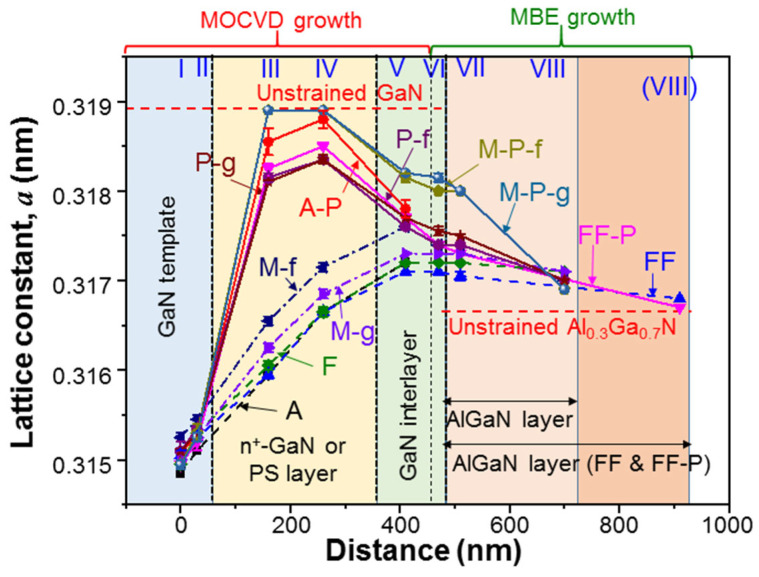
Average lattice constants, *a*, at the five or eight locations in various samples. The distance in the abscissa is defined by setting location I at zero distance. The error bars are also shown.

In row 2 of Table 2, we show the AlGaN layer thicknesses in nm estimated from TEM images for the AlGaN samples under study. Except samples FF and FF-P, all AlGaN layer thicknesses are close to 250 nm. The AlGaN layer thicknesses in samples FF and FF-P are quite close to 450 nm. The fluctuations in AlGaN thickness among those samples can be caused by non-uniform AlGaN growth over the sample surface, calibration inaccuracy, growth instability, and the variation in strain condition. The last factor can be quite important in samples XXX-f, in which AlGaN layers are overgrown after the mesa structures and/or PSs are fabricated. Different strain conditions in the templates can lead to different thicknesses in AlGaN overgrowth. However, the similar thicknesses of the AlGaN layers among different samples lay the foundation for comparing their strain-relaxation behaviors. Although the d-spacing analysis can also provide us with data of the lattice constant *c* along the c-axis, we will pay more attention to the results of the lattice constant *a* along the a-axis since it directly manifests the strain condition of a crystal layer. Rows 3–10 in Table 2 show the average lattice constants *a* at the five or eight locations in each sample under study. The error range based on the two sets of data from two different cross-sectional positions in each sample is also shown. Those data, including the error ranges, are graphically demonstrated in Figure 8. Here, in the abscissa, we define the zero distance at location I of d-spacing analysis. Each sample was grown with MOCVD in the distance range of 0–460 nm. Between 460 and 930 nm in distance, samples FF and FF-P were grown with MBE. In other AlGaN samples, MBE growth covered the distance range of 460–730 nm. It is noted that the curves for samples M-P-f and M-P-g coincide with each other except the data points at location VI. The upper and lower horizontal dashed lines indicate the lattice constants, *a*, in unstrained GaN and Al_0.3_Ga_0.7_N, at 0.3189 and 0.3166 nm, respectively [29].

To understand the variation trends in Figure 8 or Table 2, we need to keep in mind that the GaN layers are compressively strained by the sapphire substrate. The AlGaN layer is strained by the thick GaN layers. In this study, we are mainly concerned with the strain variation in the AlGaN layer when the compressive strains in the GaN layers are partially or fully relaxed. In Figure 8, we can see the increasing trend of the in-plane lattice constant, *a*, along the defined distance in sample A, which is caused by the partial relaxation of compressive strain in the n^+^-GaN layer due to heavy Si doping [30,31]. Then, in sample A-P, the compressive strain of GaN in the PS is significantly relaxed such that lattice constant *a* is increased by a large amount. It is close to the fully relaxed condition at location IV. Next, in sample F, the bottom portion of the overgrown strained AlGaN layer is lattice matched (*a* = 0.3172 nm) with the partially relaxed GaN in the GaN interlayer. However, in the top portion of the AlGaN layer, the strain is slightly relaxed. The strain variation behaviors in samples M-f and M-g are similar to that in sample F. However, the strain relaxations of GaN are stronger due to the mesa structures. In particular, the strain relaxation of GaN in sample M-f is stronger than that in sample M-g. The stronger strain relaxations or larger lattice constants, *a*, of GaN in samples M-f and M-g, when compared with sample F, lead to stronger tensile strains in the overgrown AlGaN layers and hence stronger strain relaxations (smaller critical thicknesses), i.e., larger lattice differences between the top and bottom portions of the AlGaN layers. Next, in samples P-f and P-g, the PSs lead to even stronger relaxations of the compressive strains or larger increments of the in-plane lattice constants in the PS layers and GaN interlayers. In this situation, generally the bottom portions of the AlGaN layers are even more strongly tensile-strained and the strain relaxations (critical thicknesses) in these layers become even stronger (smaller). However, due to the strain shield effect of the PS, the AlGaN layer can effectively counteract against the stress from the GaN interlayer such that its own tensile strain is reduced. In this situation, the critical thickness of the AlGaN layer is increased. If the overgrown AlGaN layer is thinner than the increased critical thickness, surface cracking would not occur. Nevertheless, a thinner overgrown AlGaN layer results in a weaker counteraction effect and hence a smaller critical thickness increment. To avoid surface cracking, the AlGaN thickness needs to be optimized. It is noted that because of the strong compressive strain from the thick sapphire substrate, the lattice constant *a* in a deep layer of the GaN template is smaller than that of un-strained Al_0.3_Ga_0.7_N. In other words, if no n^+^-GaN layer or PS existed to relax the GaN strain, the AlGaN layer could also be compressively strained by the GaN template.

By combining the strain relaxation effects of mesa structure and PS in samples M-P-f and M-P-g, the compressive strains of GaN in the PS layers become fully relaxed such that the unstrained *a* value at 0.3189 nm is observed. Under this condition, again, the stress counteractions of the AlGaN layers against the GaN interlayers make their lattice constants *a* significantly smaller than those in the PS layers. The strain behavior in sample FF is expected to be similar to that in sample F. However, the thicker overgrown AlGaN layer (~450 nm) in sample FF can more strongly counteract the tensile stress from the underlying GaN layer to further reduce the tensile strain in AlGaN. With a PS in sample FF-P, the strong strain relaxation in GaN and the thicker AlGaN layer result in an even stronger counteraction to make the difference in lattice constant *a* between the PS and GaN interlayer even larger, when compared with all other AlGaN samples with PSs. From rows 8 and 9 in Table 2, we can see that the a-axis lattice constants near the top of the GaN interlayer and the bottom of the AlGaN layer are either identical or differ by only a small amount (≤10^−4^ nm), indicating the pseudomorphic AlGaN overgrowth.

**Figure 9:**
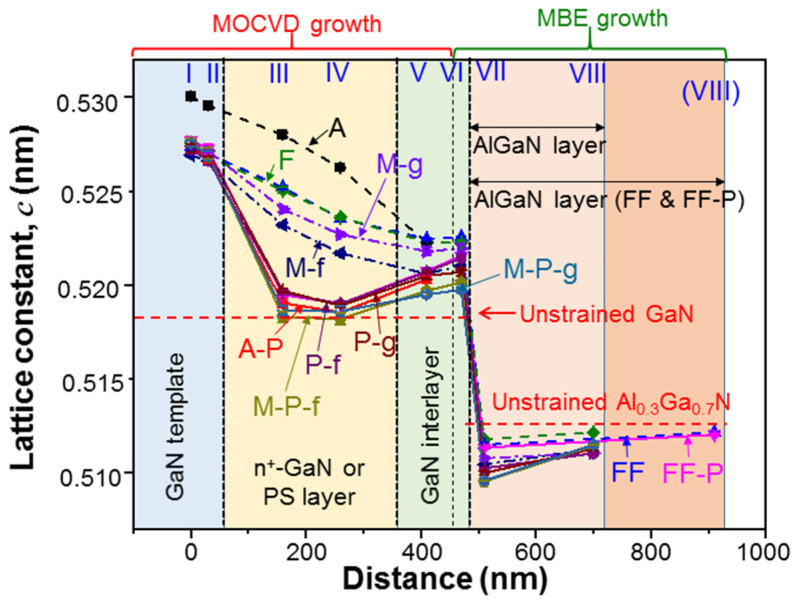
Results of the average lattice constants, *c*, at the five or eight locations in various samples similar to Figure 8.

Table 3 shows the average lattice constants *c* and the associated error ranges at the five or eight locations in each sample under study. These data, including the error ranges, are graphically demonstrated in Figure 9. Generally, a larger *a* value corresponds to a smaller *c* value. In a GaN PS layer, the strong strain relaxation makes lattice constant *c* close to the unstrained value of GaN (0.5185 nm). Near the top surfaces of the AlGaN layers in certain samples, lattice constants *c* are also close to the unstrained value of Al_03_Ga_07_N (0.5123 nm) [29]. Although the variation in lattice constant *a* is continuous from the GaN interlayer to the AlGaN layer due to the pseudomorphic overgrowth (see Figure 8), a dramatic change in lattice constant *c* can be clearly seen. Such a large difference in *c* between GaN and AlGaN makes the differentiation of the AlGaN feature from the GaN feature in a (0002)-plane XRD measurement simple, as shown in Figure 2. Although the error ranges in *c* are generally larger than those in *a*, it is difficult to see the error bars in Figure 9 because of the large variation range in *c* in the ordinate.

## 5. Discussion

In the second row from the bottom of Table 2, we show the maximum lattice constant *a* of GaN, which is designated as *a*_GaN(max)_, at those locations of d-spacing analysis. For those samples with PSs, they appear in the PS layers. For those samples without PSs, they appear in the MOCVD-grown portions of the GaN interlayers. In the bottom row of Table 2, we show the values of *a*_GaN(max)_ − *a*_GaN(VI)_, where *a*_GaN(VI)_ is the GaN lattice constant *a* at location VI in a sample. The value of *a*_GaN(VI)_ determines the AlGaN lattice constant *a* at its initial growth stage. The value of *a*_GaN(max)_ − *a*_GaN(VI)_ in a sample can be used to indicate the strain shield capability of a PS or an n^+^-GaN layer. With the strain shield effect, the lattice constants of the GaN interlayers can be effectively influenced by the overgrown AlGaN layers. In other words, the strain in an overgrown AlGaN layer can be effectively reduced. Here, we can see that in a sample without PS, the value of *a*_GaN(max)_ − *a*_GaN(VI)_ is either zero or very small. However, with a PS in a sample, the value of *a*_GaN(max)_ − *a*_GaN(VI)_ becomes significantly larger. Between the two sample series of XXX-f and XXX-g, as shown in the bottom row of Table 2, the strain shield effect caused either by a PS or by a mesa pattern is relatively stronger in the sample series of XXX-f. Therefore, by fabricating a PS or a mesa pattern before AlGaN overgrowth, we can achieve a stronger strain shield effect. In other words, AlGaN overgrowth on a strain-damped GaN template can lead to a more weakly strained AlGaN layer. It is noted that between samples P-f and P-g (M-P-f and M-P-g), due to their almost fully-relaxed conditions caused by the PSs, the strain variation behaviors of AlGaN are about the same.

**Figure 10:**
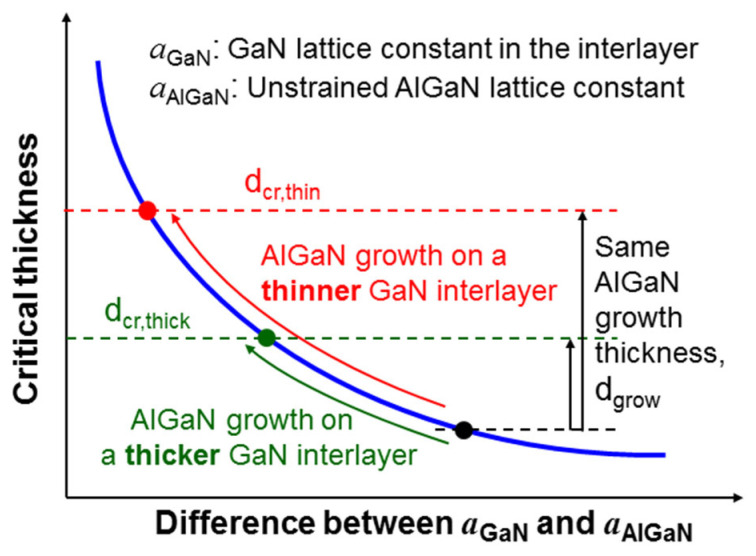
Schematic illustration for the relation between the critical thickness of the overgrown AlGaN and the thickness of the GaN interlayer.

With the strain shield effect of a PS, the GaN interlayer is essentially stress-isolated from the thick GaN template below the PS. In its initial growth stage, AlGaN is lattice matched with the top portion of the GaN interlayer. When the overgrown AlGaN becomes thicker, it counteracts against the tensile stress from the GaN interlayer such that the in-plane lattice sizes in the GaN interlayer and the AlGaN layer become closer to the unstrained AlGaN lattice constant, leading to a larger critical thickness of AlGaN. The result of this stress counteraction depends on the thickness of the GaN interlayer. In Figure 10, we schematically illustrate the relation between the AlGaN critical thickness and the GaN interlayer thickness. On a GaN template of a given lattice constant, the AlGaN critical thickness decreases with increasing Al content, as described by the blue curve in Figure 10 [2]. The increase in Al content is equivalent to the increase in lattice mismatch between AlGaN and GaN, i.e., the difference between *a*_GaN_ and *a*_AlGaN_, in the abscissa of Figure 10. Here, *a*_GaN_ is the GaN lattice constant *a* in the interlayer and *a*_AlGaN_ is the unstrained lattice constant *a* of AlGaN in the current study. As overgrown AlGaN becomes thicker, the stress counteraction reduces the difference between *a*_GaN_ and *a*_AlGaN_ such that the strain condition traces leftward along the blue curve in Figure 10 from the initial point of overgrowth (represented by the black dot) to the stop point (green or red dots). With the same AlGaN overgrowth thickness, d_grow_, the trace stops at the green (red) dot to give a smaller (larger) critical thickness, d_cr,thick_ (d_cr,thin_) when the GaN interlayer is relatively thicker (thinner). Therefore, if the GaN interlayer is thin, the final critical thickness, d_cr,thin_, can be larger than the overgrowth thickness, d_grow_, such that the overgrown AlGaN layer can be crack-free. In other words, if the tensile stress from the GaN interlayer can be overcome before the critical thickness is reached, the cracking behavior can be avoided. In this study, cracking lines on the top surfaces in all the AlGaN overgrowth samples with PSs are observed [see Figure 4(b1) and Figure 5a as examples], indicating that the GaN interlayer thickness (~120 nm) in the current study is too large to achieve the stress-overcome condition mentioned above. It is believed that a thinner GaN interlayer can avoid AlGaN surface cracking. Nevertheless, if the GaN interlayer is too thin, high-quality AlGaN overgrowth can become difficult. This is so because during the high-temperature AlGaN overgrowth, the PS below can be deformed [see Figure 4(b2)] and hence the GaN interlayer may crack if it is too thin. In this situation, the crystal quality of the early-stage AlGaN overgrowth can be poor and hence the growth of a thick layer is needed to achieve high-quality AlGaN.

Besides the aforementioned effect of enhancing the AlGaN critical thickness, the relaxation of the compressive strain in the GaN template through PS fabrication can be used to control the polarization-induced p-type behavior of an overgrown Al-decreasing AlGaN layer [32,33]. The hole distribution in such an Al-decreasing AlGaN layer is mainly caused by the spontaneous polarization gradient induced by the Al-concentration variation along the c-axis. However, the strain-induced piezoelectric polarization condition in the AlGaN layer can also greatly influence the hole distribution and p-type behavior. By relaxing the strain in the GaN template, the tensile strain distribution in the overgrown Al-decreasing AlGaN layer can be controlled for tailoring the required p-type behavior. In particular, if the PS can be laterally varied [34], a lateral variation of p-type behavior can be implemented for novel device applications [35].

## 6. Conclusions

In summary, by using d-spacing crystal lattice analysis, we have compared the lattice constant variations in different growth layers among nine samples of overgrown AlGaN on GaN templates under various strain-relaxation conditions. In particular, we observed the behavior of tensile strain reduction in AlGaN when a PS was fabricated in a GaN layer. This strain reduction in AlGaN was caused by the effective counteraction of AlGaN against the stress from the GaN interlayer, which was stress-isolated from the thick GaN template by the strain-damping PS. With the strain reduction, the critical thickness of AlGaN was increased. It is expected that AlGaN surface cracking can be avoided if the GaN interlayer right above the PS is sufficiently thin such that a strong stress counteraction can lead to a large critical thickness that is larger than the AlGaN overgrowth thickness.

## Figures and Tables

**Table 1:** Results of lattice constants *a* and *c* at the eight locations of d-spacing analysis (I–VIII) in the two regions [(1) and (2)] of TEM observations for sample M-P-f. The averages and error ranges of the results in the two regions are also shown.

Location	Composition	Lattice Constant *a* (×10^−4^ nm)			Lattice Constant *c* (×10^−4^ nm)		
		M-P-f (1)	M-P-f (2)	Average	M-P-f (1)	M-P-f (2)	Average
I	GaN template	3150	3149	3149.5 ± 0.5	5274	5278	5276.0 ± 2.0
II	GaN template	3154	3152	3153.0 ± 1.0	5267	5273	5270.0 ± 3.0
III	Porous structure (PS)	3189	3189	3189.0 ± 0.0	5186	5180	5183.0 ± 3.0
IV	Porous structure (PS)	3189	3189	3189.0 ± 0.0	5185	5179	5182.0 ± 3.0
V	GaN interlayer (MOCVD)	3181	3182	3181.5 ± 0.5	5198	5196	5197.0 ± 1.0
VI	GaN interlayer (MBE)	3180	3180	3180.0 ± 0.0	5202	5201	5201.5 ± 0.5
VII	AlGaN overgrown layer	3180	3180	3180.0 ± 0.0	5096	5095	5095.5 ± 0.5
VIII	AlGaN overgrown layer	3169	3169	3169.0 ± 0.0	5114	5115	5114.5 ± 0.5

**Table 2:** Row 2: Thicknesses of the AlGaN layers in various samples in nm. Rows 3–10: Average lattice constants, *a* (×10^−4^ nm), at the five or eight locations in various samples. Row 11: Maximum GaN lattice constants, *a*. Row 12: Differences between the maximum GaN lattice constants *a* and those for GaN at location VI. The error ranges of *a* values are also shown.

Sample	A	A-P	F	M-f	M-g	P-f	P-g	M-P-f	M-P-g	FF	FF-P
AlGaN thickness	---	---	242.9	244.4	252.0	264.4	250.7	253.0	248.5	455.7	462.9
I (u-GaN template)	3148.5 ±0.0	3151.0 ±0.0	3149.5 ±0.5	3152.5 ±0.5	3150.0 ±0.0	3151.0 ±1.0	3151.0 ±0.0	3149.5 ±0.5	3149.5 ±0.5	3150.5 ±0.5	3149.5 ±0.5
II (u-GaN template)	3151.0 ±0.0	3152.5 ±0.5	3153.0 ±0.0	3154.5 ±0.5	3152.5 ±0.5	3153.5 ±0.5	3153.0 ±1.0	3153.0 ±1.0	3152.5 ±0.5	3152.5 ±0.5	3152.0 ±1.0
III (n^+^-GaN or PS)	3159.5 ±0.5	3185.5 ±1.5	3160.5 ±0.5	3165.5 ±0.5	3162.5 ±0.5	3181.5 ±0.5	3181.0 ±0.0	3189.0 ±0.0	3189.0 ±0.0	3159.5 ±0.5	3182.5 ±0.5
IV (n^+^-GaN or PS)	3166.5 ±0.5	3188.0 ±1.0	3166.5 ±0.5	3171.5 ±0.5	3168.5 ±0.5	3183.5 ±0.5	3183.5 ±0.5	3189.0 ±0.0	3189.0 ±0.0	3166.5 ±0.5	3185.0 ±0.0
V (GaN-MOCVD)	3172.0 ±0.0	3178.0 ±1.0	3172.0 ±0.0	3176.0 ±0.0	3173.0 ±0.0	3176.0 ±0.0	3177.0 ±0.0	3181.5 ±0.5	3182.0 ±0.0	3171.0 ±0.0	3177.0 ±0.0
VI (GaN-MBE)	---	---	3172.0 ±0.0	3174.0 ±0.0	3173.0 ±0.0	3174.0 ±0.0	3175.5 ±0.5	3180.0 ±0.0	3181.5 ±0.5	3171.0 ±0.0	3174.0 ±0.0
VII (AlGaN layer)	---	---	3172.0 ±0.0	3174.0 ±0.0	3173.0 ±0.0	3174.0 ±0.0	3175.0 ±0.0	3180.0 ±0.0	3180.0 ±0.0	3170.5 ±0.5	3173.0 ±0.0
VIII (AlGaN (layer)	---	---	3171.0 ±0.0	3170.0 ±0.0	3171.0 ±0.0	3170.0 ±0.0	3170.0 ±0.0	3169.0 ±0.0	3169.0 ±0.0	3168.0 ±0.0	3167.0 ±0.0
*a*_GaN(max)_	---	---	3172.0 ±0.0	3176.0 ±0.0	3173.0 ±0.0	3183.5 ±0.5	3183.5 ±0.5	3189.0 ±0.0	3189.0 ±0.0	3171.0 ±0.0	3185.0 ±0.0
*a*_GaN(max)_ − *a*_GaN(VI)_	---	---	0.0 ± 0.0	2.0 ± 0.0	0.0 ± 0.0	9.5 ± 0.5	8.0 ± 1.0	9.0 ± 0.0	7.5 ± 0.5	0.0 ± 0.0	11.0 ± 0.0

**Table 3:** Average lattice constants, *c* (×10^−4^ nm), at the five or eight locations in various samples. The error ranges of *c* values are also shown.

Sample	A	A-P	F	M-f	M-g	P-f	P-g	M-P-f	M-P-g	FF	FF-P
I (u-GaN template)	5300.5 ±1.5	5273.5 ±1.5	5275.5 ±1.5	5269.0 ±1.0	5276.0 ±0.0	5272.5 ±2.5	5273.0 ±2.0	5276.0 ±2.0	5275.5 ±1.5	5273.5 ±1.5	5277.0 ±2.0
II (u-GaN template)	5295.5 ±0.5	5267.0 ±2.0	5268.5 ±0.5	5266.0 ±2.0	5271.5 ±0.5	5266.5 ±2.5	5270.5 ±2.5	5270.0 ±3.0	5269.0 ±2.5	5270.0 ±0.5	5272.5 ±2.5
III (n^+^-GaN or PS)	5280.0 ±1.0	5190.5 ±2.5	5250.5 ±1.5	5232.0 ±1.0	5240.5 ±1.5	5196.0 ±1.0	5197.0 ±1.0	5183.0 ±3.0	5186.5 ±2.5	5252.0 ±1.0	5195.0 ±1.0
IV (n^+^-GaN or PS)	5262.5 ±0.5	5185.5 ±2.5	5236.0 ±1.0	5217.0 ±1.0	5227.0 ±1.0	5190.0 ±1.0	5189.0 ±1.0	5182.0 ±3.0	5186.0 ±3.0	5235.5 ±1.5	5190.0 ±1.0
V (GaN-MOCVD)	5222.5 ±0.5	5203.0 ±2.0	5223.0 ±0.0	5206.0 ±0.0	5218.0 ±2.0	5207.0 ±1.0	5205.0 ±2.0	5197.0 ±1.0	5195.0 ±1.0	5224.5 ±0.5	5207.5 ±0.5
VI (GaN-MBE)	---	---	5222.5 ±0.5	5210.5 ±0.5	5219.5 ±2.5	5214.5 ±0.5	5207.0 ±2.0	5201.5 ±0.5	5197.5 ±1.5	5225.5 ±0.5	5215.5 ±1.5
VII (AlGaN layer)	---	---	5118.0 ±0.0	5104.5 ±0.5	5108.0 ±1.0	5102.5 ±0.5	5100.0 ±1.0	5095.5 ±0.5	5096.0 ±1.0	5115.0 ±1.0	5113.5 ±0.5
VIII (AlGaN (layer)	---	---	5121.5 ±0.5	5113.0 ±1.0	5110.5 ±0.5	5110.5 ±0.5	5113.0 ±1.0	5114.5 ±0.5	5115.0 ±1.0	5121.5 ±0.5	5120.5 ±0.5

## Data Availability

All the data supporting reported results can be found in the text of this paper.
